# Rescue liver re-transplantation after graft loss due to severe rejection in the setting of pre-transplant nivolumab therapy

**DOI:** 10.1007/s12328-021-01521-4

**Published:** 2021-10-13

**Authors:** Yalda Dehghan, Gabriel T. Schnickel, Mojgan Hosseini, Adam M. Burgoyne, Veeral H. Ajmera, Gerald P. Morris, Michel H. Mendler, Justin R. Parekh, Farid Abushamat, Irine Vodkin, Yuko Kono

**Affiliations:** 1 Division of Gastroenterology and Hepatology, Department of Medicine, University of California San Diego, La Jolla, CA USA; 2 Department of Surgery, Division of Transplant and Hepatobiliary Surgery, University of California San Diego School of Medicine, La Jolla, CA USA; 3 Department of Pathology, University of California San Diego, La Jolla, CA USA; 4 Division of Hematology-Oncology, Department of Medicine, Moores Cancer Center, University of California San Diego, La Jolla, CA USA

**Keywords:** Liver transplantation, Acute rejection, Malignancy, Immune regulation, Hepatology

## Abstract

Immune checkpoint inhibitors (ICI) have been used to treat hepatocellular carcinoma (HCC) since 2017. The safety of ICIs in the setting of solid organ transplantation remains controversial. When used in the post-transplant setting, ICIs have been associated with high allograft rejection rates, but there are few published reports on the use of ICIs prior to transplant. We present the first reported case of rescue liver re-transplantation after loss of the first allograft due to severe acute rejection with extensive hepatic necrosis in the setting of pre-transplant ICI therapy with the PD-1 inhibitor nivolumab. It is likely that the durable immune response triggered by nivolumab contributes to graft rejection, therefore extreme caution should be taken when using ICIs before transplant until further investigation has been conducted on their safety in the pre-transplant setting.

## Introduction

Systemic therapy options for advanced hepatocellular carcinoma (HCC) have expanded substantially with the advent of immune checkpoint inhibitors (ICIs). The programmed cell death protein-ligand 1 (PD-L1) inhibitor atezolizumab is now approved as first-line treatment (in combination with bevacizumab), and the PD-1 inhibitors such as nivolumab are approved for second-line treatment [1, 2]. Given their efficacy, there is growing interest in using ICIs as neoadjuvant or adjuvant therapy.

Ultimately, liver transplantation is a curative treatment for HCC. Most transplant centers employ pre-transplant locoregional therapies to prevent tumor progression and keep patients within transplant-eligible criteria or to down-stage tumors to gain transplant eligibility. Older systemic therapies such as the multikinase inhibitor sorafenib failed to show efficacy as a neoadjuvant therapy before liver transplantation, with minimal response rates and associated intolerability [3].

Although ICIs are increasingly used to treat HCC, their safety in the setting of solid organ transplantation remains unknown. Previous reviews have found that ICI therapy was associated with high allograft rejection rates when used in patients with prior solid organ transplants [4, 5]. However, few studies have focused on the effects of ICIs in the neoadjuvant setting before solid organ transplant. Current reports demonstrate variable outcomes, and therefore the use of ICIs for this purpose remains controversial. We present the first reported case of a successful re-transplantation after the initial allograft was lost due to severe acute rejection with subtotal necrosis in a patient who had received nivolumab prior to transplantation.

## Case report

A 60-year-old woman with HCV cirrhosis was diagnosed with UNOS-OPTN stage T2 HCC (2.5 cm LI-RADS 5 lesion and a 1.0 cm LI-RADS 3 lesion) on MRI. She underwent transarterial chemoembolization and microwave ablation. Repeat MRI demonstrated viable tumor and she started treatment with sorafenib and nivolumab with repeat microwave ablation. She was then listed for orthotopic liver transplant (OLT). Sorafenib was discontinued after 3 months due to intolerance, but nivolumab was continued; she received 240 mg every 2 weeks the first month, then 480 mg every 4 weeks for 15 months, at which time MRI showed two nonviable treated lesions. She underwent OLT 5 weeks after discontinuing nivolumab. She remained well compensated with an MELD Na of 11.

**Fig. 1:**
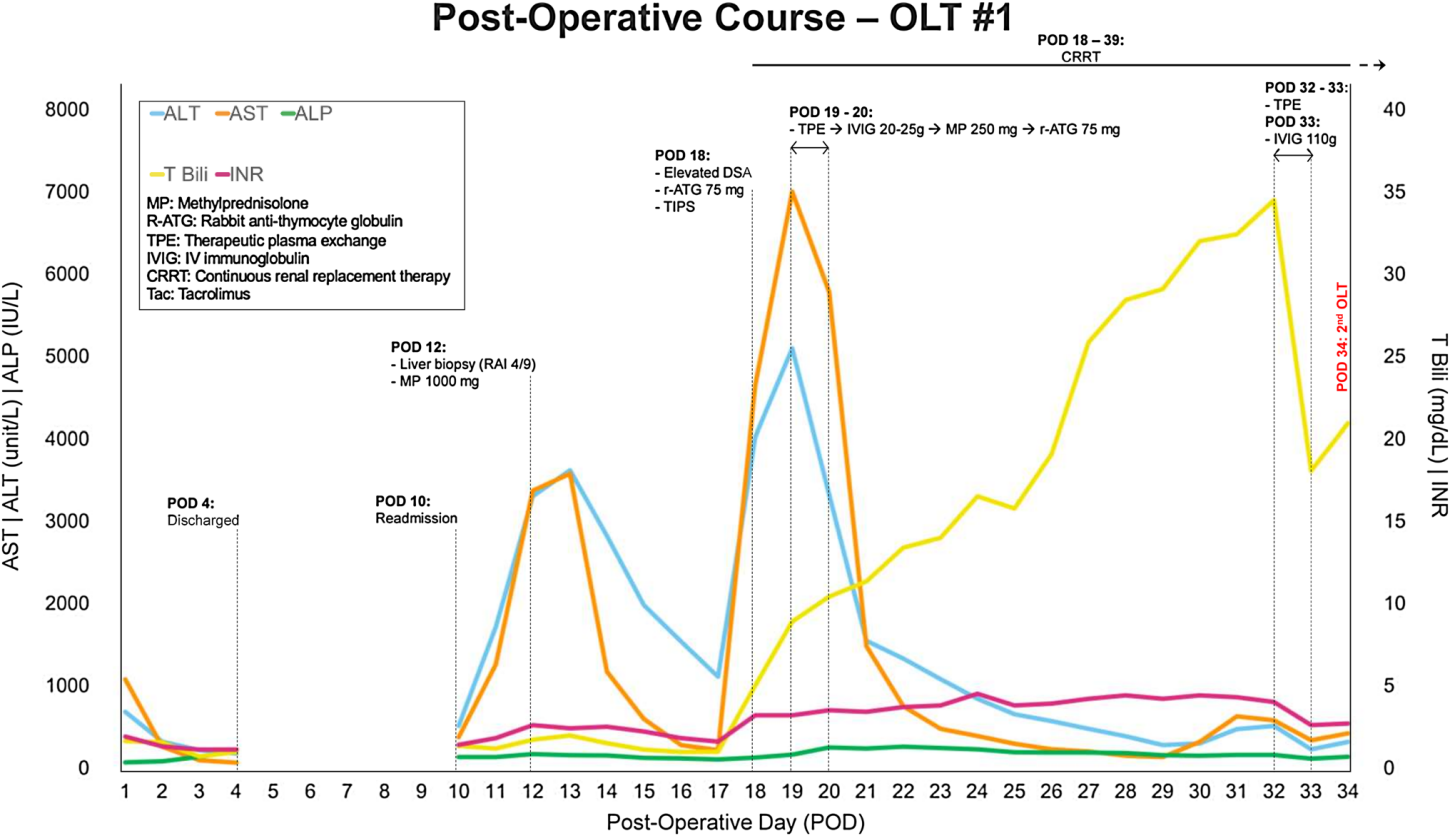
Post-operative course for the first OLT with immunosuppression regimen and rejection management. *AST* aspartate aminotransferase, *ALT* alanine aminotransferase, *ALP* alkaline phosphatase, *T Bili* total bilirubin, *INR* international normalized ratio, *MP* methylprednisolone, *R-ATG* rabbit anti-thymocyte globulin, *TPE* therapeutic plasma exchange, *IVIG* IV immunoglobulin, *CRRT* continuous renal replacement therapy, *Tac* tacrolimus, *RAI* rejection activity index, *TIPS* transjugular intrahepatic portosystemic shunt, *DSA* donor-specific antibodies

The patient received an ABO-compatible liver from a donation after brain death (DBD) donor with a history of IV drug use (negative for HIV, HCV, and HBV). Cold and warm ischemic times were 3 h, 9 min and 26 min, respectively. OLT was performed using piggyback technique. The explanted native liver demonstrated a 0.8 cm post-treatment well-differentiated HCC in segment 8, and two necrotic nodules in segment 8 without evidence of residual tumor or vascular invasion*.* Post-operative course from the first OLT is summarized in Fig. 1. Post-operative ultrasound demonstrated patent vasculature with normal waveforms and no evidence of retrograde flow. She received standard immunosuppression with steroid taper, tacrolimus, and mycophenolate mofetil (MMF). The immediate post-operative course was uneventful, and she was discharged on post-operative day (POD) 4.

**Fig. 2:**
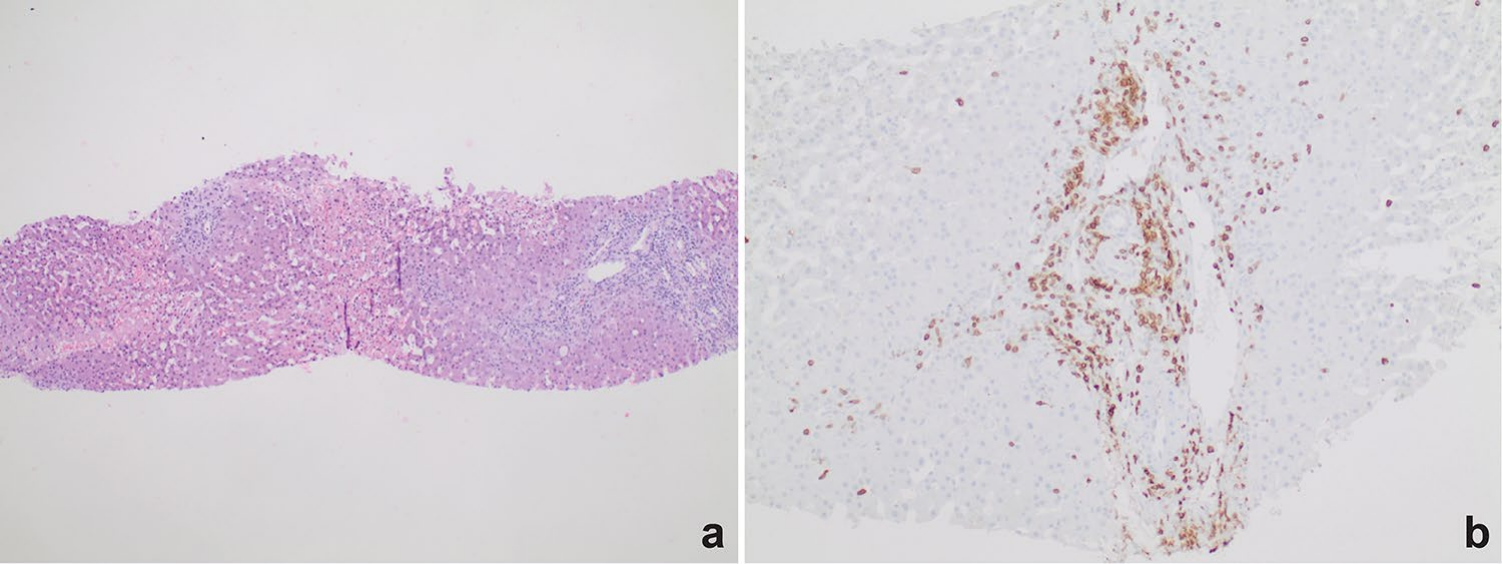
The first allograft biopsy. **a** Sub-massive hepatic necrosis (centrilobular and bridging) with predominantly portal lymphocytic inflammation and endothelitis. Rejection activity index (RAI) 4/9. **b** First allograft biopsy, CD3 immunostain. Lymphocytic infiltrate primarily comprised of CD3 + , CD8 + T cells

On POD 10, the patient was readmitted for fever and transaminases in the 300 s. Doppler ultrasound and CT were concerning for venous outflow obstruction and reversed portal venous flow. A hepatic venogram revealed mild right hepatic vein stenosis and a venoplasty was performed; there was no evidence of anastomotic stenosis or occlusive thrombus. Balloon-occluded retrograde transvenous obliteration of a large splenorenal shunt was performed to improve hepatic inflow. Transjugular liver biopsy demonstrated acute cellular rejection with sub-massive hepatic necrosis involving 60% of the core with moderate lymphocytic portal inflammation and a rejection activity index (RAI) of 4/9. The lymphocytic infiltrate primarily comprised CD3 lymphocytes with minimal CD20-positive B cells (Fig. 2). PDL-1 immunostain was positive in only rare inflammatory cells (< 1%), and PD-1 demonstrated intermediate staining (< 5% of inflammatory cells). Treatment with methylprednisolone 1000 mg was initiated on POD 12 and transaminases down-trended. On POD 18 multiple donor specific antibodies (DSA) against HLA class II antigens were detected. Antibodies against DR8 and DQ7 demonstrated significant prozone inhibition in neat serum with high levels of antibodies detectable at 1:10 serum dilution. Despite 75 mg anti-thymocyte globulin (ATG), transaminases increased to the 4000 s with INR 3.2 and bilirubin 8.9. Ultrasound demonstrated non-occlusive portal vein thrombosis and a venogram revealed a portosystemic gradient of 33 mmHg despite widely patent hepatic vein anastomosis. Due to concern that this was secondary to massive necrosis or swelling within the liver, transjugular intrahepatic portosystemic shunt (TIPS) with thrombolysis was performed to prevent complete thrombosis of the portomesenteric system and enable re-transplant. Given the DSA and biopsy findings, she was concurrently treated for antibody-mediated and acute cellular rejection. Therapeutic plasma exchange (TPE) was initiated on POD 19 and she received IV immunoglobulin (IVIG) followed by methylprednisolone and ATG for 2 days. Following TPE × 2, IVIG × 2 and ATG × 3, DSA were still present. Given lack of improvement and low likelihood of graft recovery, she was re-listed for liver transplant with a biologic MELD Na greater than 40. By POD 23, she had developed grade III hepatic encephalopathy with worsening coagulopathy. On POD 31, a donor liver became available but was not selected due to continuously elevated DR8 and DQ7 antibodies that would likely cross-react with the donor organ. Daily TPE was started on POD 31 to decrease DSA and methylprednisolone taper was continued.

**Fig. 3:**
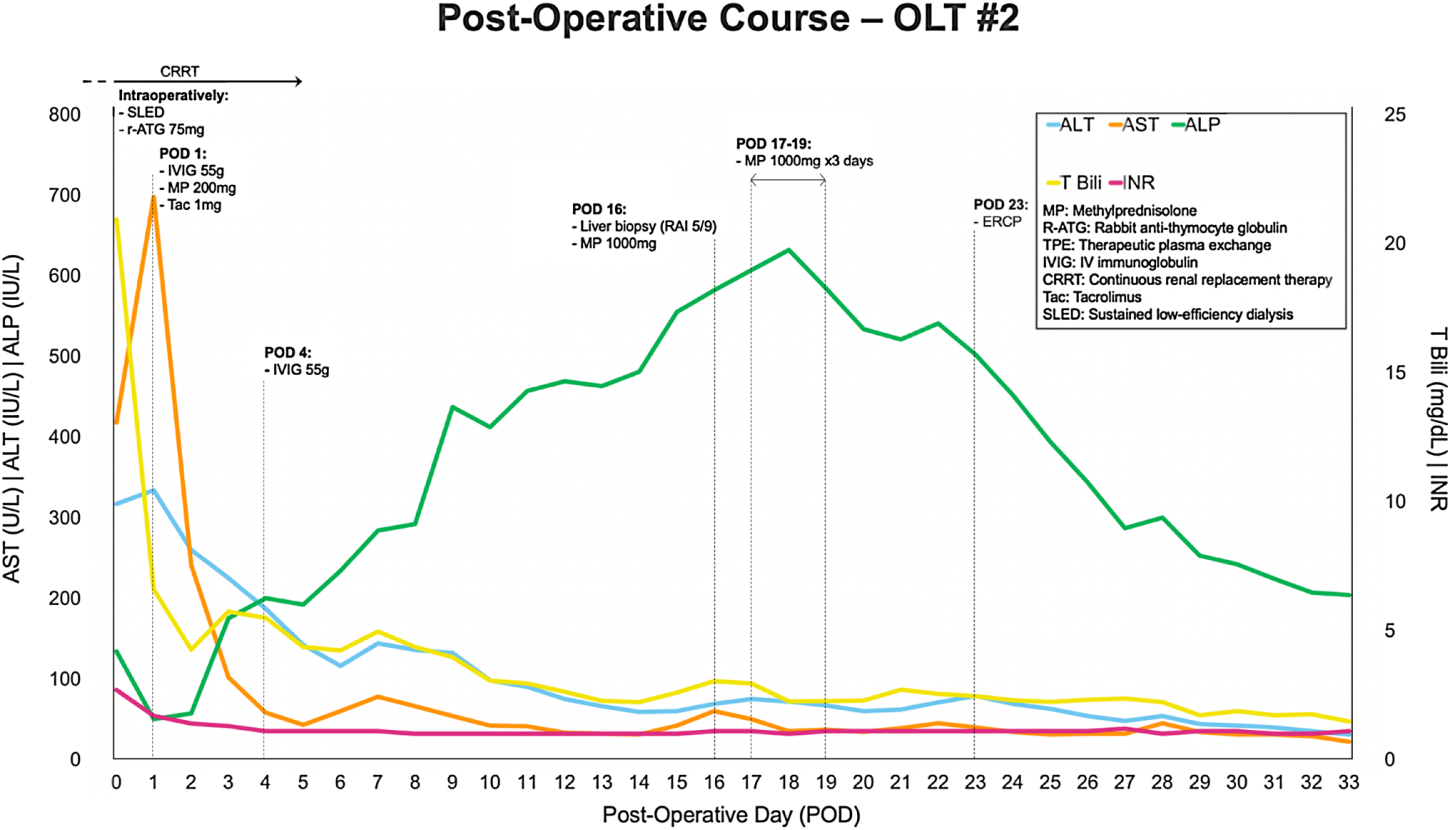
Post-operative course for the seconf OLT with immunosuppression regimen and rejection management. *AST* aspartate aminotransferase, *ALT* alanine aminotransferase, *ALP* alkaline phosphatase, *T Bili* total bilirubin, *INR* international normalized ratio, *MP* methylprednisolone, *R-ATG* rabbit anti-thymocyte globulin, *TPE* therapeutic plasma exchange, *IVIG* IV immunoglobulin, *CRRT* continuous renal replacement therapy, *Tac* tacrolimus, *SLED* sustained low-efficiency dialysis, *ERCP* endoscopic retrograde cholangiopancreatography, *RAI* rejection activity index

**Fig. 4:**
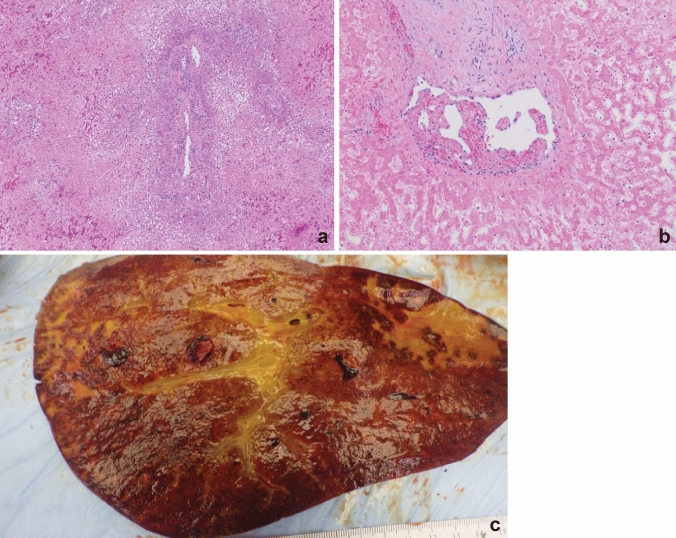
Explanted allograft. **a** Explanted allograft with massive hepatic necrosis surrounding a portal tract. Few surviving hepatocytes are steatotic. **b** Centrizonal necrosis and organizing thrombus with recanalization of the central vein. **c** Gross specimen demonstrating massive necrosis with hemorrhage and vascular thrombosis

On POD 34, she underwent re-transplant from a DBD, ABO-compatible donor. Virtual cross-matching was performed; donors with DSA present at a 1:10 dilution were avoided. She was given IVIG the day before transplant and received intraoperative ATG. Cold and warm ischemic times were 4 h, 37 min and 33 min, respectively. The post-operative course from her second OLT is summarized in Fig. 3. Explant of the previous allograft revealed massive hepatic necrosis with hemorrhage and organizing portal vein thrombi (Fig. 4). Biopsies of the newly transplanted liver demonstrated mild preservation injury with minimal steatosis, rare necrosis, and mild lobular inflammation.

She required massive transfusion during surgery due to profound coagulopathy and required multiple vasopressors post-operatively. DSA on POD 1 was negative. She received IVIG on POD 1 and 4. Methylprednisolone taper and tacrolimus were started on POD 1 and MMF was added on POD 19 after resolution of thrombocytopenia and leukopenia. On POD 9, ALP acutely increased to the 400 s while DSA remained negative. A biopsy demonstrated acute cellular rejection (RAI 5/9) with mixed portal inflammation, bile duct injury, lobular inflammation, and hepatocyte necrosis. She was given a 1000 mg methylprednisolone bolus for 3 days with taper. Endoscopic retrograde cholangiopancreatography (ERCP) performed on POD 23 demonstrated anastomotic stricture, and a plastic stent was placed, which was later replaced with a metal stent. She was discharged on POD 33 and continues to do well at 18 months post-transplantation.

## Discussion

We present a patient who was successfully re-transplanted after demonstrating severe acute rejection with massive hepatic necrosis and loss of the first allograft, which we attribute to immune-mediated damage from nivolumab use prior to initial transplant.

ICIs have revolutionized cancer treatment partly due to the durability of tumor responses, which can persist for years. Studies in tumor types for which ICI data is more mature (i.e., melanoma) have shown tumor regression for several months following ICI discontinuation [6], with no evidence of recurrence in 86% of patients 18 months after cessation of therapy [7]. Despite their wide clinical use, the exact mechanisms of action of anti-PD/anti-PD-L1 and anti-CTLA4 antibodies are not fully understood. Anti-PD1 antibodies have not been shown to exhibit dose-dependent efficacy or toxicity [8]. Furthermore, target occupancy of PD-1 persists for much longer than the drug half-life, with PD-1 saturation demonstrated on circulating lymphocytes for up to 100 days after a single 10-mg/kg dose of nivolumab, which has a 20-day serum half-life at that dose [9].

The potential for permanent immune reprogramming and the ability to produce a long-lasting immune response have made the use of PD-1 inhibitors in the setting of transplantation controversial. PD-1 inhibitors have been associated with high rates of allograft rejection in solid organ transplant recipients (SOTRs) [4, 5]. A recent systematic review evaluated the rates of allograft rejection in SOTRs and reported a rejection rate of 52.2% in SOTRs who received nivolumab [10]. Other studies have demonstrated an association between acute rejection and positive PD-L1 staining in allografts of patients who experienced acute rejection after nivolumab therapy [11, 12]. A recent review conducted by Nguyen et al. studied the incidence of organ rejection in SOTRs who had received ICI therapy and found that all reported rejections were T-cell mediated rejections, while 21.4% were also characterized as antibody-mediated rejections [13]. Biopsies from our patient demonstrated rare (< 1% of inflammatory cells) staining of PD-L1 with intermediate (< 4% of inflammatory cells) staining of PD-1. Given the durable response triggered by ICI therapy (which can persist even after cessation of therapy), there are likely permanent effects on the immune milieu after checkpoint inhibition that may or may not correlate with PD-1/PD-L1 expression [6]. Our case demonstrated similar clinical characteristics to reports of acute allograft rejection in SOTRs treated with nivolumab and raises concerns regarding the safety of ICI therapy in the pre-transplant setting.

While ICI therapy in the post-transplant setting appears to carry an increased risk of allograft rejection, few reports have focused on pre-transplant ICI therapy. Nordness et al. described a case of fatal rejection when nivolumab was discontinued only 8 days before transplant, while Schwacha-Eipper et al. described a case of successful transplantation with no evidence of rejection when nivolumab treatment was discontinued 6 weeks before transplantation [14, 15]. Our patient demonstrated severe acute rejection with hepatic necrosis and vascular thrombosis despite discontinuing nivolumab 5 weeks prior to transplant. This pattern of rejection is similar to the case described by Nordness et al. While there have been reports of centrilobular confluent necrosis in the biopsies of patients with nivolumab-induced hepatotoxicity and suggestions that PD-1/PD-L1 blockade can lead to hepatotoxicity, the fulminant hepatic necrosis demonstrated in our patient is less common [16, 17]. Although vascular complications and thromboses are potential consequences of acute rejection and were consistent with the pattern of rejection described by Nordness et al., it should be noted that this patient’s acute rejection of the allograft could potentially be secondary to vascular complications. A venoplasty was performed for right hepatic vein stenosis when the patient was admitted on POD 10 after the first OLT; however, the stenosis was likely due to hepatic swelling as there was no evidence of anastomotic complications or thrombus.

The successful re-transplant in this case was attributed to the use of pre-operative TPE, IVIG and ATG to reduce DSA and significantly lower immune response. It should be noted that in this patient with stage T2 HCC, the systemic treatment was initiated and continued by a community physician prior to listing and was not part of routine HCC management guidelines. This case also demonstrates the value of evaluating anti-HLA antibodies before transplant. In the systematic review conducted by Nguyen et al. which primarily focused on patients who received post-transplant ICI therapy, it was found that while all reported rejections were T cell-mediated rejections, 21.4% were also characterized as antibody mediated. It is worth noting that in many of these reports, specific data regarding the presence of DSA was not available. In our case, there was no pre-transplant antibody testing before the first OLT, so it is unknown if there was pre-existing DSA, however this information was used before the second transplant to identify more suitable donors by avoiding those with DSA present at 1:10 dilution. The DSA were significantly reduced before re-transplantation, which we believe contributed to a favorable outcome. The methods employed in this case to reduce DSA may guide further studies that investigate the safety of ICI therapy pre-transplant, while also helping to better characterize the observed patterns of rejection in patients who receive pre-transplant ICI therapy.

As ICIs increasingly are used for HCC treatment, it is vital to better characterize their safety in liver transplantation. Atezolizumab (anti-PD-L1) and bevacizumab (anti-VEGF) combination therapy is now a first-line regimen for advanced HCC and as a result, more patients with advanced HCC may be down-staged with ICIs and become eligible for transplants. Currently, most of the data concerning the safety of using ICIs for down-staging are limited to independent case reports, therefore we recommend further investigation with a carefully designed clinical trial protocol to determine the safety of this approach prior to use in the liver transplant population. Given the multiple reports of graft failure and fatality, providers should exercise extreme caution in the use of ICIs for down-staging outside of a research protocol, as safety remains unclear.

## Abbreviations

ALP: Alkaline phosphatase
ATG: Anti-thymocyte globulin
CT: Computerized tomography
CTLA4: Cytotoxic T-lymphocyte-associated antigen 4
DBD: Donation after brain death
DSA: Donor-specific antibodies
ERCP: Endoscopic retrograde cholangiopancreatography
HCC: Hepatocellular carcinoma
HBV: Hepatitis B virus
HCV: Hepatitis C virus
HIV: Human immunodeficiency virus
HLA: Human leukocyte antigen
ICI: Immune checkpoint inhibitor
INR: International normalized ratio
IV: Intravenous
IVIG: Intravenous immunoglobulin
LI-RADS: Liver imaging reporting and data system
MELD: Model for end-stage liver disease
MRI: Magnetic resonance imaging
MMF: Mycophenolate mofetil
OLT: Orthotopic liver transplant
PD-1: Programmed cell death protein 1
PD-L1: Programmed death-ligand 1
POD #: Post-operative day #
RAI: Rejection activity index
SOTRs: Solid organ transplant recipients
TIPS: Transjugular intrahepatic portosystemic shunt
TPE: Therapeutic plasma exchange
UNOS-OPTN: United Network for Organ Sharing-Organ Procurement and Transplantation Network
VEGF: Vascular endothelial growth factor
